# Metastatic pathway-specific transcriptome analysis identifies *MFSD4* as a putative tumor suppressor and biomarker for hepatic metastasis in patients with gastric cancer

**DOI:** 10.18632/oncotarget.7269

**Published:** 2016-02-08

**Authors:** Mitsuro Kanda, Dai Shimizu, Haruyoshi Tanaka, Masahiro Shibata, Naoki Iwata, Masamichi Hayashi, Daisuke Kobayashi, Chie Tanaka, Suguru Yamada, Tsutomu Fujii, Goro Nakayama, Hiroyuki Sugimoto, Masahiko Koike, Michitaka Fujiwara, Yasuhiro Kodera

**Affiliations:** ^1^ Department of Gastroenterological Surgery (Surgery II), Nagoya University Graduate School of Medicine, Nagoya, Japan

**Keywords:** gastric cancer, hepatic metastasis, MFSD4, biomarker, tumor suppressor

## Abstract

Gastric cancer (GC) with hepatic metastasis remains a fatal disease. Global expression profiling was conducted using tissues from patients who had GC with synchronous hepatic metastasis, and major facilitator superfamily domain containing 4 *(MFSD4)* was identified as a candidate biomarker for hepatic metastasis in GC. Functional and expression analyses of this molecule in GC cell lines and clinical samples were conducted. We analyzed *MFSD4* expression, DNA methylation, and copy number. RNA interference experiments evaluated the effects of *MFSD4* expression on cell phenotype and apoptosis. We analyzed tissues of 200 patients with GC to assess the diagnostic performance of *MFSD4* levels for predicting hepatic recurrence, metastasis, or both. Differential expression of *MFSD4* mRNA by GC cell lines correlated positively with the levels of *NUDT13* and *OCLN* mRNAs and inversely with those of *BMP2*. Hypermethylation of the *MFSD4* promoter was detected in cells with lower levels of *MFSD4* mRNA. Inhibition of *MFSD4* expression significantly increased the invasiveness and motility of GC cells but did not influence cell proliferation or apoptosis. *MFSD4* mRNA levels in primary GC tissues were reduced in patients with concomitant hepatic metastasis or recurrence compared with those without. Low levels of *MFSD4* mRNA in primary GC tissues were an independent risk factor of hepatic recurrence and metastasis. *MFSD4* expression in gastric tissues may represent a useful biomarker for identification of patients at high risk for hepatic recurrence, metastasis, or both.

## INTRODUCTION

Gastric cancer (GC) is one of the most common malignancies and the third leading cause of cancer-related deaths worldwide [1, 2]. Hepatic metastasis and relapse contribute to the high incidence of GC-related fatalities, and represent a frequent and crucial problem for oncologists [3, 4]. Metastasis is a multistep process involving detachment from a primary site, invasion of surrounding connective tissue, transmigration across the basement membrane, intravasation, formation of tumor emboli, extravasation, and colonization of target organs [5, 6]. GC metastasizes through three distinct metastatic pathways; lymphatic, hematogeneous and direct dissemination from the serosal surface [7-9]. Each metastatic pathway is considered to require survival of select clones that accumulate specific mutations or epigenetic abnormalities. Thus, knowledge of the unique molecular attributes of each metastatic pathway will improve our understanding of the biology of metastatic GC and facilitate development of specific biomarkers and appropriate targeted therapies, leading to efficacious personalized treatment [10]. Studies to identify and explore diagnostic biomarkers and molecular targets to predict and treat hepatic metastases among others are warranted.

To achieve this goal, we conducted transcriptome analysis using a next-generation sequencing platform and identified major facilitator superfamily domain containing 4 (*MFSD4*) as a candidate biomarker for hepatic metastasis of GC. In the current study, we conducted expression and functional analyses of *MFSD4* to validate our global expression profiling data. To our knowledge this is the first study that evaluated significance of *MFSD4* in the metastatic process of GC.

## RESULTS

### Identification of *MFSD4* as an inhibitor of hepatic metastasis

Transcriptome analysis identified 21 candidate genes (Supplementary Table 1). After literature review regarding current knowledge on functional aspect of each gene, we decided to focus on *MFSD4* because 1) *MFSD4* exhibited the second strongest suppression in primary GC tissues among 21 candidates (approximately 5% of the corresponding noncancerous adjacent gastric mucosa), 2) *MFSD4* expression levels were comparable between primary GC tissues and liver tissues with metastases, 3) *MFSD4* encodes a membrane trafficking protein that may be involved in cellular functions and transportation of growth factors, and 4) we sought novel molecules as potential biomarkers for GC there have been no reports on oncological roles of *MFSD4*.

### *MFSD4* mRNA levels of GC cell lines

**Figure 1:**
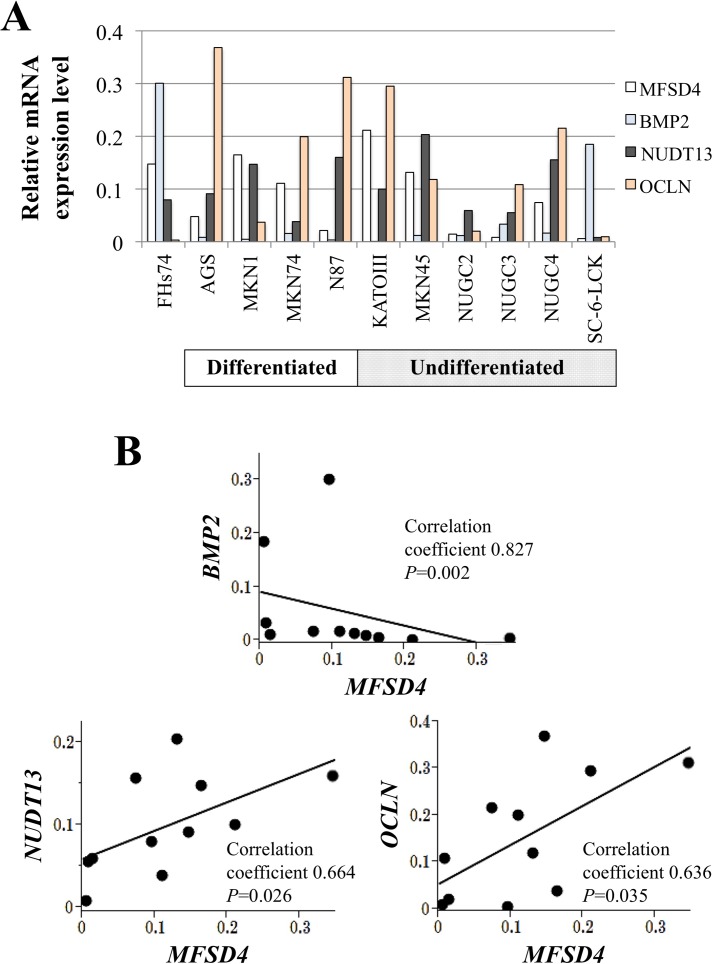
Expression analysis of 10 GC cell lines and the nontumorigenic epithelial cell line FHs74 **A.** Expression of *MFSD4* and genes expressed at similar differential levels were identified using PCR array analysis. **B.** Analysis of the correlation between the mRNA levels of *MFSD4* and those of *BMP2*, *NUDT13*, and *OCLN*.

The levels of *MFSD4* mRNAs differed among GC cell lines. AGS, N87, NUGC2, NUGC3, and SC-6-JCK cells expressed *MFSD4* mRNA at levels that were approximately < 50% of that of the control cell line FHs74 (Figure 1A). *MFSD4* mRNA levels did not differ according to the degree of differentiation of the GC cells. PCR array analysis revealed that mRNAs encoding nudix-type motif 13 (*NUDT13*) and occludin (*OCLN*) were expressed at levels that correlated significantly with those of the mRNA encoding *MFSD4*, whereas the expression levels of bone morphogenetic protein 2 (*BMP2*) mRNA correlated inversely with those of *MFSD4* (Figure 1A and 1B).

### Mechanism of suppression of *MFSD4* transcription in GC

**Figure 2:**
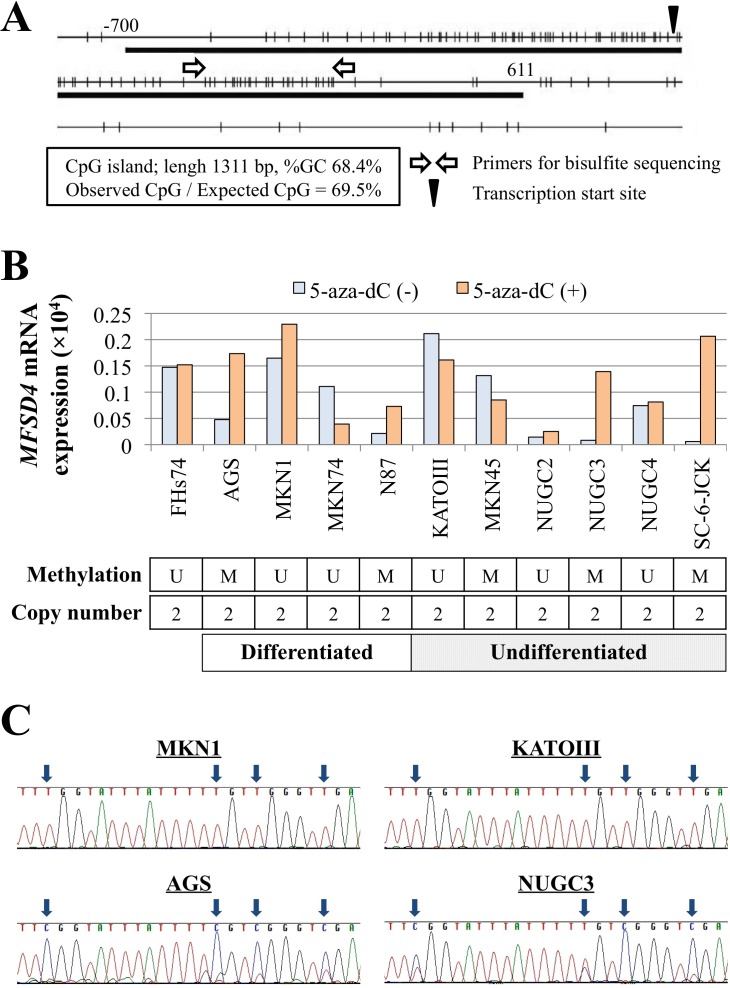
Expression, methylation and gene copy-number analysis **A.** A CpG island was detected near the *MFSD4* transcription-initiation site, extending upstream into the promoter region. **B.** *MFSD4* mRNA levels in 11 cell lines before or after 5-aza-dC treatment. The methylation status of the *MFSD4* promoter and the copy number of the *MFSD4* locus are shown and summarized in the lower boxes. M, methylated; U, unmethylated. **C.** Representative bisulfite sequencing data. All CpG sites in AGS cell were retained as CG and those of MKN1 and KATOIII cells were converted to TG. NUGC3 cell showed partial methylation.

Identification of a CpG island in the *MFSD4* promoter region (Figure 2A) suggested that hypermethylation of the CpG islands inhibits *MFSD4* transcription in GC cells. Bisulfite sequencing analysis revealed that the *MFSD4* promoter was methylated in AGS, N87, MKN45, NUGC3, and SC-6-JCK cells. When we compared the levels of *MFSD4* mRNAs in GC cell lines before and after demethylation, reactivation of *MFSD4* expression was detected in cells with promoter hypermethylation (Figure 2B). Representative results of direct sequence analysis are shown in Figure 2C. Moreover, the copy number of the *MFSD4* locus of each GC cell line was the same as that of the controls (Figure 2B).

### Effect of *MFSD4* knockdown on the malignant phenotype and apoptosis of GC cells

**Figure 3:**
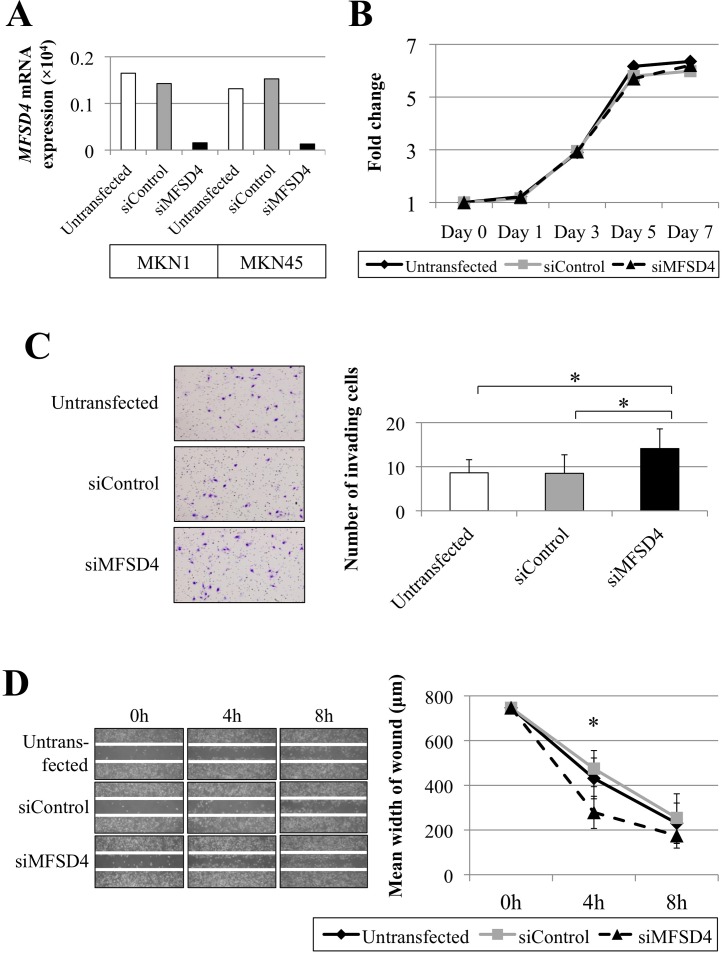
Effects of siRNA-mediated knockdown of *MFSD4* expression on GC cells **A.** siRNA-mediated *MFSD4* knockdown was determined using qPCR. **B.** Cell proliferation assay. The *MFSD4* siRNA had little effect on the proliferation of MKN1 cells. **C.** Cell invasion assays. The number of invading cells was significantly increased by knockdown of *MFSD4* expression. *p < 0.05. **D.** Wound-healing cell migration assays. Inhibition of *MFSD4* expression significantly increased the migration of MKN1 cells. *p < 0.05.

Inhibition of *MFSD4* expression using a specific siRNA was conducted to evaluate the function of *MFSD4* in GC cells. The effect of *MFSD4* knockdown was confirmed using qPCR assays (Figure 3A). We evaluated the proliferation, invasion, migration, and apoptosis of MKN1 (differentiated type) and MKN45 (undifferentiated type) cells, which produced relatively high levels of *MFSD4* mRNA. The proliferation rates of these cell lines were not influenced by the inhibition of *MFSD4* expression (Figure 3B). In contrast, knockdown of *MFSD4* expression significantly increased invasion (Figure 3C) and migration (Figure 3D) of MKN1 cells compared with the untransfected and siControl-transfected cells. The size of the population of apoptotic cells was not altered by knockdown of *MFSD4* expression (Supplementary Figure 1). The results for MKN45 cells were similar to those for MKN1 (data not shown).

### Clinical implications of *MFSD4* mRNA levels in primary GC tissues

**Figure 4:**
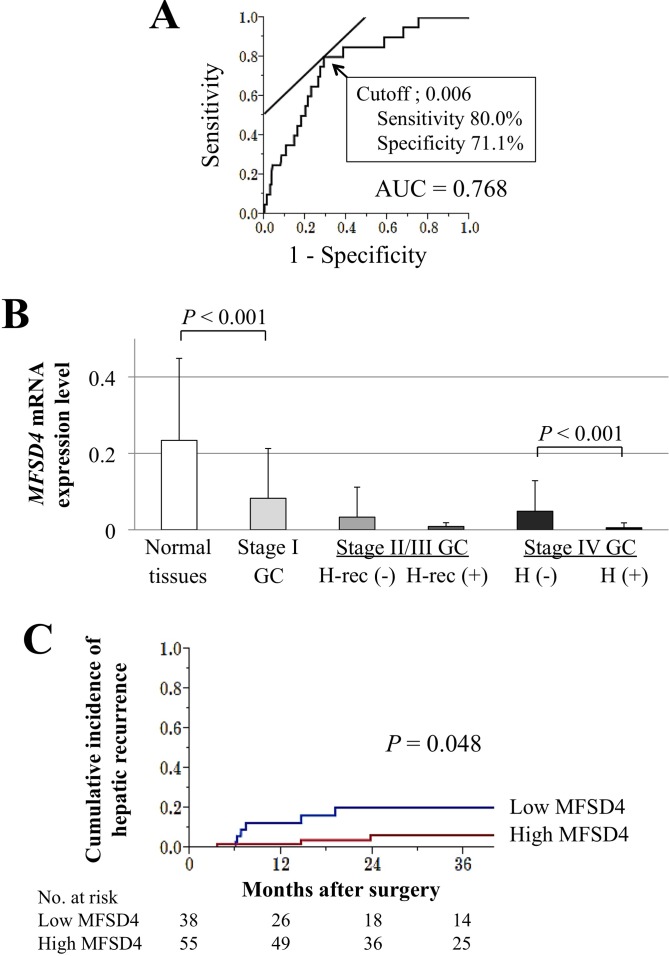
Clinical implications of the levels of *MFSD4* mRNA in GC tissues **A.** ROC curve analysis of the value of *MFSD4* expression levels for predicting hepatic metastasis. The optimal cutoff value = 0.006. **B.** The levels of *MFSD4* mRNA in the corresponding adjacent noncancerous tissues and GC tissues according to disease stage and recurrence patterns. **C.** The cumulative incidence of hepatic recurrence was significantly higher in the low-*MFSD4* group when focusing on 93 patients with stage II/III GC. GC, gastric cancer; AUC, area under the curve.

In 177 (88.5%) patients, *MFSD4* mRNA expression levels were lower in GC tissues compared to the corresponding noncancerous adjacent tissues. The diagnostic performance of the detection of hepatic metastasis was correlated with the levels of *MFSD4* expression in primary GC tissues. There were 11 patients with synchronous hepatic metastasis and nine patients who experienced hepatic recurrences after curative gastrectomy. The AUC value of *MFSD4* expression levels was 0.768 for detection of synchronous hepatic metastasis or hepatic recurrence within 2 years after surgery, and the optimal cutoff value = 0.006 (sensitivity = 80.0%, specificity = 71.1%, Figure 4A). A significant decrease of *MFSD4* expression was detected in patients with stage I GC compared with the noncancerous adjacent tissues. The levels of *MFSD4* expression were lower in patients with stage II/III GC who experienced hepatic recurrences following curative gastrectomy compared with those without hepatic recurrences, although the difference was not statistically significant. In patients harboring distant metastasis at the time of surgery (stage IV), the levels of *MFSD4* expression in primary GC tissues were significantly lower in patients with synchronous hepatic metastasis compared with those without (Figure 4B).

When patients were classified into high and low *MFSD4* expression groups according to the proposed cutoff (*MFSD4* mRNA level = 0.006), the cumulative incidence of findings that indicated recurrences in the form of hepatic metastases were significantly higher in the low *MFSD4* group (*p* = 0.048, Figure 4C). *MFSD4* mRNA levels were independent of tumor depth, differentiation, and disease stage whereas they were significantly associated with macroscopic type, infiltrative growth type, and metastatic sites (peritoneal or liver) (Table 1). To investigate further the relationship between high *MFSD4* mRNA levels in GC tissues and hepatic metastasis, multivariate binomial logistic analysis was conducted and revealed that low *MFSD4* levels were an independent risk factor for hepatic metastasis, recurrence, or both (odds ratio 5.28, 95% confidence interval 1.63-20.6, *p* = 0.005; Table 2). The *MFSD4* mRNA levels were not significantly associated with overall survival of the 200 enrolled patients (Supplementary Figure 2A), or overall (Supplementary Figure 2B) and disease-free survival (Supplementary Figure 2C) of patients with stage II/III GC.

**Table 1:** Association between *MFSD4* mRNA levels and clinicopathological characteristics of 200 patients with gastric cancer

Variables	High *MFSD4*	Low *MFSD4*	*P*
Age < 65 year ≥ 65 year	55 74	24 47	0.219
Sex Male Female	88 41	53 18	0.337
CEA (ng/ml) ≤ 5 > 5	104 25	51 20	0.159
CA19-9 (IU/ml) ≤ 37 > 37	104 25	52 19	0.232
Tumor location Entire Upper third Middle third Lower third	15 28 38 48	4 16 19 32	0.438
Tumor size (mm) < 50 ≥ 50	50 79	23 48	0.369
Macroscopic type Borrmann type 4/5 Others	31 98	5 66	0.002*
Tumor depth (UICC) pT1-3 pT4	55 74	39 32	0.096
Differentiation Differentiated Undifferentiated	44 85	30 41	0.255
Lymphatic involvement Absent Present	17 112	9 62	0.919
Vessel invasion Absent Present	51 78	22 49	0.227
Infiltrative growth type Invasive growth Expansive growth	62 67	16 55	<0.001*
Lymph node metastasis Absent Present	37 92	20 51	0.939
Peritoneal lavage cytology Negative Positive	86 43	59 12	0.011*
Synchronous liver metastasis Absent Present	128 1	61 10	<0.001*
UICC stage I II III IV	24 17 38 50	8 16 22 25	0.246

* Statistically significant (*p* < 0.05). CEA, carcinoembryonic antigen; CA19-9, carbohydrate antigen 19-9; UICC, Union for International Cancer Control

**Table 2:** Predictive factors of hepatic metastasis/recurrence for 200 patients with gastric cancer

Variables		H/ H-rec (−)	H/ H-rec (+)	Univariate		Multivariate		
				OR	*P*	OR	95%CI	*P*
Age	< 65 year ≥ 65 year	73 107	6 14	1.59	0.352			
Sex	Male Female	123 57	18 2	4.17	0.027	3.28	0.73 - 23.9	0.128
CEA	≤ 5 ng/ml > 5 ng/ml	144 36	11 9	3.27	0.018	2.51	0.78 - 8.13	0.122
CA19-9	≤ 37 IU/ml > 37 IU/ml	143 37	13 7	2.08	0.159			
Tumor location	Lower third Others	71 109	9 11	0.80	0.632			
Tumor size	< 50 mm ≥ 50 mm	68 112	5 15	1.82	0.248			
Macroscopic type	Borrmann type 4 Others	35 145	1 20	4.59	0.070			
Tumor depth	pT1-3 pT4	82 98	12 8	1.79	0.219			
Differentiation	Differentiated Undifferentiated	63 117	9 11	2.27	0.085			
Lymphatic involvement	Absent Present	25 155	1 19	3.06	0.212			
Vessel invasion	Absent Present	72 108	1 19	12.7	<0.001	14.4	2.62 - 269	<0.001*
Infiltrative growth	Invasive Expansive	77 103	1 19	14.2	<0.001	6.00	0.99 - 116	0.050
Lymph node metastasis	Absent Present	54 126	3 17	2.43	0.136			
Peritoneal lavage cytology	Negative Positive	127 53	18 2	3.76	0.044	2.80	0.59 - 20.6	0.204
*MFSD4* expression	High Low	125 55	4 16	9.09	<0.001	5.28	1.63 - 20.6	0.005*

* Statistically significant in multivariate analysis (*p*<0.05). H/ H-rec, hepatic metastasis/recurrence; OR, odds ratio; CI, confidence interval; CEA, carcinoembryonic antigen; CA19-9, carbohydrate antigen 19-9; UICC, Union for International Cancer Control.

## DISCUSSION

Here we conducted a transcriptome analysis to explore metastatic-pathway specific molecules associated with GC and identified *MFSD4* as a candidate tumor suppressor and inhibitor of hepatic metastasis. *MFSD4* resides on human chromosome 1q32.1 and is predicted to encode the multi-pass transmembrane protein *MFSD4* comprising 514 amino acid residues (56 kDa) [11, 12]. Although the *MFSD4* paralog KIA1919 is involved in glucose transport and is ubiquitously expressed, its biological function is still under investigation [13].

In GC cell lines, *MFSD4* mRNA was expressed at different levels independent of each cell's differentiated phenotype. EMT is a crucial process for cancer cells to increase the ability to migrate and invade, which are required for the development of metastatic lesions [6, 14]. We found that *NUDT13* and *OCLN* were expressed in concert with *MFSD4*, and the expression levels of *BMP2* correlated inversely with those of *MFSD4* in GC cells lines. *BMP2* increases the invasiveness of cells *via* activation of matrix metalloproteinase-2, which is required during the dispersal stage of the EMT [15-18]. *NUDT13* is a mitochondrial enzyme, and its expression decreases during the EMT, although its function is unknown [19]. *OCLN*, a 65-kDa tetraspan integral membrane protein, contributes to the stabilization of the intercellular tight junctions and optimal barrier function and is considered an inhibitor of the EMT [20, 21]. In the absence of published data on the biological function of *MFSD4*, our data implicate that *MFSD4* participates in the process of EMT through its coordinate expression with other EMT-regulating molecules in GC.

Promoter hypermethylation leads to the transcriptional silencing of tumor suppressor genes [22-24]. We show here that *MFSD4*-promoter hypermethylation was frequently accompanied by inhibition of *MFSD4* transcription. Further, *MFSD4* mRNA levels increased in cells treated with a DNA methylation inhibitor. These findings indicate that promoter hypermethylation is a pivotal mechanism that inhibits *MFSD4* transcription. To the best of our knowledge, this is the first report of the hypermethylation of *MFSD4*. In contrast, *MFSD4* expression was not reactivated after de-methylation in MKN45 and NUGC2 cells. We assume that other mechanisms inhibit *MFSD4* transcription, such as LOH, because the *MFSD4* locus resides within a known hotspot of chromosomal alterations. However, there was no detectable difference in *MFSD4* copy numbers among the GC cell lines. Other regulatory mechanisms, such as histone modification and point mutations, might be also responsible for suppression of *MFSD4* in some GC cells. Further, we show here that inhibition of *MFSD4* expression contributed to the migration and invasion of GC cell lines but not to proliferation and apoptosis. These findings suggested that suppression of *MFSD4* plays a crucial role in tumor progression and highlighted its usefulness as a potential therapeutic target in GC.

In surgically-resected gastric tissues, 88.5% of patients had lower *MFSD4* mRNA expression levels in GC tissues compared to the corresponding noncancerous adjacent tissues, and decreased *MFSD4* expression was found at the earliest stage of GC. These findings indicated that *MFSD4* may play a tumor suppressor-like role in pathogenesis of GC. Our findings that the levels of *MFSD4* mRNA in GC tissues serve as an independent risk factor for hepatic recurrence and metastasis indicates the potential of *MFSD4* expression as a novel diagnostic or predictive biomarker for hepatic metastasis in patients with GC. Evaluation of *MFSD4* expression may be useful for preoperative staging, postoperative monitoring, and selection of optimal multimodal strategy. For example, physicians may be able to stratify patients at risk for hepatic metastasis at the time of endoscopic biopsy or surgical resection of the primary GC, enabling them to provide appropriate management to treat concurrent or future hepatic metastasis. Moreover, the value of *MFSD4* expression for the prognosis of hepatic metastasis will be enhanced by the development of assays to detect *MFSD4* expression or methylation in serum samples because of the use of a relatively noninvasive technique that can be frequently repeated. Interestingly, the expression of *MFSD4* had no significant association with vessel invasion, which is known as a typical risk factor for hepatic metastasis. This finding may highlight more the utility of *MFSD4* for stratifying patients at risk of hepatic metastasis independent of vessel invasion. On the other hand, *MFSD4* expression had no association with lymph node metastasis, though it was significantly associated with positive lavage cytology, which meant that *MFSD4* may be involved also in formation of peritoneal metastasis for some extent. Possible reasons that *MFSD4* expression had no association with lymph node metastasis are given as follows. In this study, only presence or absence of lymph node metastasis was evaluated regardless of the number and location of the metastatic nodes. Since GC frequently metastases to the regional lymph nodes even in the earlier stage of the disease, the presence of lymph node metastasis may not always reflect the aggressiveness of GC. Moreover, each metastatic pathway has different underlying molecular mechanisms and suppression of *MFSD4* may facilitate hepatic (hematogenous) metastasis without influencing on lymphatic metastasis.

The present study has certain limitations. First, extensive expression analyses of proteins that potentially interact with *MFSD4* must be conducted to further understand the biological functions of *MFSD4* in GC. Second, this study was limited by the relatively small sample size and lack of external validation of the reproducibility of the expression assays and their standardization across laboratories. Finally, better understanding of the tumor-suppressive functions of *MFSD4* can be potentially gained by the enforced expression of *MFSD4* in mouse xenograft models.

Nevertheless, taken together, our findings indicate that decreased *MFSD4* expression was associated with hepatic metastasis and that *MFSD4* acts as a tumor suppressor in GC by inhibiting the malignant phenotype of cancer cells. *MFSD4* expression in gastric tissues represents a promising biomarker for identification of patients at high risk for hepatic metastasis.

## MATERIALS AND METHODS

### Transcriptome analysis

Surgically resected gastric and liver specimens of four patients with GC and synchronous hepatic metastasis were subjected to transcriptome analysis. The four patients had one or two metastatic lesions confined in the unilateral robe of the liver but no other distant metastases (e.g. lung, bone, brain, peritoneal and distant lymph nodes) and received the sufficient information necessary to give informed consent before surgery. Global expression profiling was conducted using the HiSeq platform (Illumina, San Diego, CA) to compare the expression levels of 57761 genes among the primary GC tissues, the corresponding noncancerous adjacent gastric mucosae, and hepatic metastases. In the current study, we decided to focus on *MFSD4* which was identified as one of candidate genes in the transcriptome analysis.

### Sample collection

Ten GC cell lines were used in this study. MKN1, MKN45, MKN74, NUGC2, NUGC3, NUGC4 and SC-6-JCK were obtained from the Japanese Collection of Research Bioresources Cell Bank (JCRB, Osaka, Japan) and AGS, KATOIII and N87 were from the American Type Culture Collection (ATCC, Manassas, VA, USA). A control, non-tumourigenic epithelial cell line (FHs74) was purchased from ATCC. The cell lines have been tested using the short tandem repeat -polymerase chain reaction method and authenticated by the JCRB Cell Bank on January 2015. Cell lines were cultured at 37°C in Dulbecco's modified Eagle's medium (DMEM; Sigma-Aldrich, St. Louis, MO, USA) supplemented with 10% fetal bovine serum (FBS) in an atmosphere containing 5% CO_2_. Primary GC tissues and the corresponding noncancerous adjacent tissues were collected from 200 patients who underwent gastric resection for GC at the Department of Gastroenterological Surgery, Nagoya University Hospital between 2001 and 2014. These samples were collected consecutively, except that samples from pretreated patients and those from early-stage cancer from which sufficient amount of samples were unavailable were excluded. The tissue samples were immediately frozen in liquid nitrogen and stored at −80°C. Relevant clinical data including clinical stages according to the 7th edition of the Union for International Cancer Control (UICC) classification system were retrieved from the prospectively compiled database at the department. The tumor infiltrative pattern has been routinely evaluated by hematoxylin and eosin stained sections as a pathological characteristic of surgically resected specimens and classified into expansive and invasive growth types according to clarity of the tumor border [25]. This study conforms to the ethical guidelines of the World Medical Association Declaration of Helsinki - Ethical Principles for Medical Research Involving Human Subjects, and written informed consent for the use of clinical samples and data, as required by the Institutional Review Board at Nagoya University, Japan, was obtained from all patients.

### Analysis of *MFSD4* mRNA levels

*MFSD4* mRNA levels were determined using a quantitative real-time reverse-transcription polymerase chain reaction (qRT-PCR) assay. Total RNAs (10 μg per sample), which were isolated from 11 cell lines and 200 primary GC tissues as well as the corresponding noncancerous adjacent tissues were used to generate complementary DNAs after a quality check by measuring the optical density to confirm that the ratio of the absorbance at 260 and 280 nm ranges from 1.8 to 2.0. The RT-PCR amplification reaction was performed as follows: Initial denaturation at 95°C for 10 min, 40 cycles at 95°C for 10 s, and at 60°C for 30 s. All samples were tested in triplicate, and samples without template were included in each PCR plate as a negative control. A SYBR-Green PCR core reagents kit (Applied Biosystems, Foster City, CA, USA) was used to perform qRT-PCR, and real-time detection of SYBR-Green fluorescence emission intensity was performed using an ABI StepOnePlus Real-Time PCR System (Applied Biosystems). The levels of glyceraldehyde-3-phosphate dehydrogenase (*GAPDH*) mRNA were quantified in each sample, and the expression level of each sample was calculated as the value of *MFSD4* mRNA divided by that of *GAPDH* mRNA [26, 27]. Primers are listed in Supplementary Table 2.

### PCR array analysis of gene expression

To analyze gene expression in 10 GC cell lines and the FHs74 cell line, we used the Human Epithelial to Mesenchymal Transition (EMT) RT^2^ Profiler PCR Array (Qiagen, Hilden, Germany) comprising 84 key genes, including those that encode proteins that function in the processes as follows: transcription, extracellular matrix formation, epithelial to mesenchymal transition (EMT), differentiation, morphogenesis, growth, proliferation, migration, cytoskeletal formation [28].

### Methylation analysis of *MFSD4*

The nucleotide sequence of the *MFSD4* promoter region was analyzed to determine the presence or absence of CpG islands defined as follows: ≥ 200-bp region of DNA with a high GC content (> 50%) and an Observed CpG/Expected CpG ratio ≥ 0.6. We used CpG Island Searcher software (http://cpgislands.usc.edu/) to determine the locations of CpG islands [29, 30]. Genomic DNAs of the cell lines were treated with bisulfite for bisulfite sequence analysis. After PCR amplification using specific primers (Supplementary Table 2), PCR products were subcloned into a TA cloning vector (Invitrogen, Carlsbad, CA, USA). The DNAs were mixed with 3 mL of a specific primer (M13) and 4 mL of Cycle Sequence Mix (ABI PRISM Terminator v1. 1 Cycle Sequencing Kit; Applied Biosystems). Sequence analysis was conducted using an Applied Biosystems ABI310, and sequence electropherograms were generated using ABI Sequence Analysis 3.0 software (Applied Biosystems). To assess the relation of promoter hypermethylation to *MFSD4* transcription, GC cells (1.5 × 10^6^) were treated with 5-aza-2′-deoxycytidine (5-aza-dC) (Sigma-Aldrich) to inhibit DNA methylation and then cultured for 6 days with medium changes on days 1, 3, and 5. RNA was extracted, and qRT-PCR was performed as described [31].

### Copy number analysis

Using purified genomic DNA obtained from cell lines, the copy number *MFSD4* was determined using TaqMan Copy Number Assays (Applied Biosystems). Genomic DNAs (20 ng per cell line) were amplified using specific primer pairs using an ABI StepOnePlus Real-Time PCR System (Applied Biosystems) according to the manufacturer's instructions. Two assays were employed as follows: upstream (assay ID: Hs01431727_cn, within the exon 1 of *MFSD4*) and downstream (assay ID: Hs00149995_cn within the exon 10 of *MFSD4*). Data were analyzed using CopyCaller Software (Life Technologies) [32].

### Knockdown of *MFSD4* expression using a small interfering RNA (siRNA)

The Accell siRNA transfection method (Dharmacon, Lafayette, CO, USA) was used to transfect an siRNA specific for *MFSD4* mRNA (si*MFSD4*) into MKN1 and MKN45 cells that expressed relatively high levels of *MFSD4* mRNA. The control siRNA was designated siControl [33]. After transfection, cells were incubated with serum-free DMEM (Sigma-Aldrich) for 72 h. The *MFSD4* mRNA levels of transfected (siControl, si*MFSD4*) and untransfected cells were determined using qPCR.

### Cell proliferation assay

Cell proliferation was evaluated using the Premix WST-1 Cell Proliferation Assay System (Takara Bio Inc., Japan). MKN1 and MKN45 cells (5 × 10^3^ cells per well) were seeded into 96-well plates in DMEM supplemented with 2% FBS and incubated for 7 days. The optical density of the solution in each well was measured on days 1, 3, 5 and 7 following the addition of 10 μl of WST-1.

### Cell invasion assay

The ability of GC cells to invade Matrigel was determined using BioCoat Matrigel invasion chambers (BD Biosciences, Bedford, MA, USA) according to the manufacturer's protocol. MKN1 and MKN45 cells (2.5 × 10^4^ cells per well) were seeded into the upper well of the chamber in serum-free DMEM. After 48 h, cells on the lower surface of the membrane were fixed, stained, and counted using a microscope (200× magnification, eight randomly selected fields).

### Wound-healing assay

The migration of GC cells was evaluated using wound-healing assays. MKN1 and MKN45 cells (2 × 10^4^ cells per well) were seeded into 12-well plates in serum-free DMEM using the ibidi Culture insert method (ibidi, Martinsried, Germany) to establish wound gaps of a defined width. After 24 h, the insert was removed, and the width of the wound as a function of time was measured at 100-μm intervals (20 per well, 40× magnification).

### Apoptosis assay

The effect of *MFSD4* knockdown on induction of apoptosis was analyzed using the FITC Annexin V/Dead Cell Apoptosis Kit with FITC annexin V and PI for Flow Cytometry (Invitrogen, Carlsbad, CA, USA) according to the manufacturer's instruction. Briefly, control, si*MFSD4*-transfected and ultraviolet-light irradiated (60 s) MKN1 cells were washed in phosphate-buffered saline and centrifuged. Cells (5 × 10^5^ cells/ml) were incubated with 5 μL of FITC annexin V and 100 ng of propidium iodide and incubated at room temperature for 15 min. The cells were analyzed using a flow cytometer (BD FACSAria Fusion; BD Biosciences, Bedford, MA, USA).

### Statistical analysis

The significance of the difference between two variables was assessed using Spearman's rank correlation coefficient. The Mann-Whitney test was used to compare the differences between two groups. Goodness-of-fit was assessed by calculating the area under the curve (AUC) of the receiver operating characteristic (ROC) curve, and the optimal cutoff value was determined using the Youden index. Associations between *MFSD4* mRNA levels and clinical variables were evaluated using Fisher's exact test or the chi-square test. Risk factors for hepatic metastasis were evaluated using binomial logistic analysis. Overall survival rates were calculated using the Kaplan-Meier method, and the difference between survival curves was analyzed using the log-rank test. All statistical analyses were performed using JMP 10 software (SAS Institute Inc., Cary, NC, USA). A value of *p* < 0.05 was considered statistically significant.

## SUPPLEMENTARY MATERIAL FIGURES AND TABLES


