# Supplementary material for: Metastatic pathway-specific transcriptome analysis identifies MFSD4 as a putative tumor suppressor and biomarker for hepatic metastasis in patients with gastric cancer
Source: Oncotarget. 2016 Feb 8;7(12):13667–79. doi: 10.18632/oncotarget.7269 (PMC4924669; doi:10.18632/oncotarget.7269)
Supplement: Supplementary file 1 [file oncotarget-07-13667-s001.pdf]

Metastatic pathway-specific transcriptome analysis identifies *MFSD4* as a putative tumor suppressor and biomarker for hepatic metastasis in patients with gastric cancer

Supplementary Material

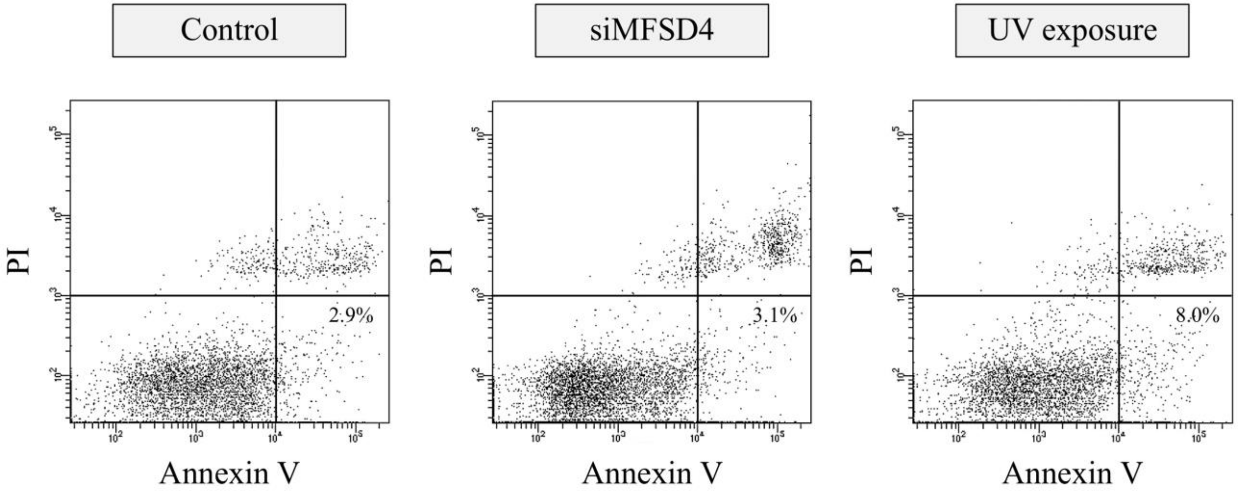

Supplementary Figure S1: Apoptosis assay. Inhibition of *MFSD4* expression had little influence on apoptosis, whereas the population of apoptotic cells was increased by exposure to ultraviolet light.

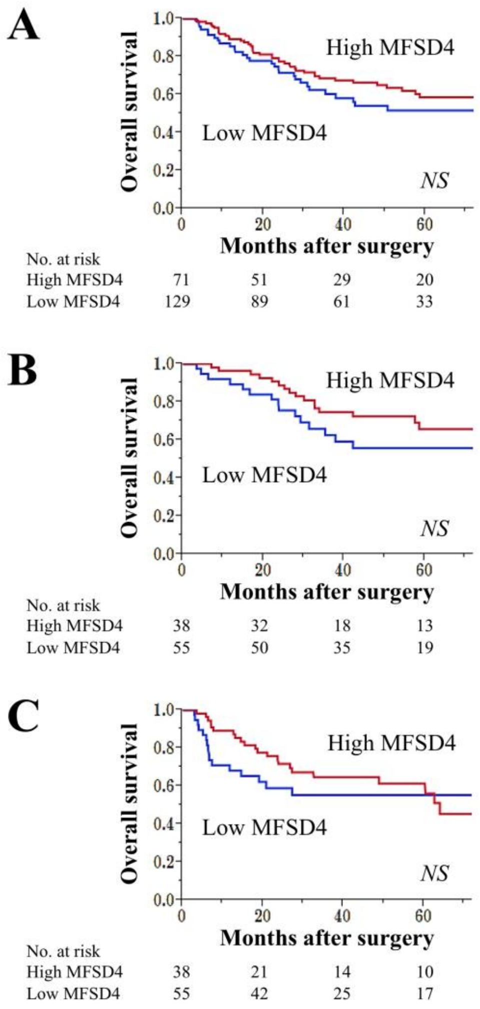

Supplementary Figure S2: Prognostic impact of *MFSD4* expression. (A) Overall survival rate of 200 patients. Overall (B) and disease-free (C) survival rate in 93 patients with stage II/III GC after curative gastrectomy. NS, not statistically significant.

Supplementary Table 1. List of candidate genes at lower levels in gastric cancer tissues from patients with hepatic metastasis

Symbol	GC tissue/Normal		Name	Location	Function	H-meta/GC tissue	
	**Log_2**	***P***				**Log_2**	***P***
GPR155	-4.43	<0.0001	G protein-coupled receptor 155	2q31.1	Multipass membrane protein	0.01	1.0000
MFSD4	-4.32	<0.0001	major facilitator superfamily domain containing 4	1q32.1	Membrane transporter	-0.69	0.4659
HRASLS2	-4.26	<0.0001	HRAS-like suppressor 2	11q12.3	Metabolic enzyme	-1.38	0.1086
SLC9A4	-3.95	<0.0001	solute carrier family 9, subfamily A, member 4	2q12.1	Signal transducer	-0.40	0.6221
ALDH3A1	-3.40	<0.0001	aldehyde dehydrogenase 3 family, member A1	17p11.2	Metabolic enzyme	-0.95	0.3085
DPT	-3.22	<0.0001	dermatopontin	1q12-q23	Extracellular matrix protein	-0.12	0.8893
POU2AF1	-2.93	<0.0001	POU class 2 associating factor 1	11q23.1	Transcription factor	-1.07	0.0575
IRF4	-2.92	<0.0001	interferon regulatory factor 4	6p25-p23	Transcription factor	-0.84	0.2034
ASAH2	-2.82	<0.0001	N-acylsphingosine amidohydrolase 2	10q11.21	Metabolic enzyme	0.09	1.0000
GSTA1	-2.76	0.0002	glutathione S-transferase alpha 1	6p12.1	Metabolic enzyme	1.17	0.0828
MZB1	-2.71	<0.0001	marginal zone B and B1 cell-specific protein	5q31.2	Immunoglobulin mediator	-1.19	0.1480
LIFR	-2.59	<0.0001	leukemia inhibitory factor receptor alpha	5p13-p12	Cytokine receptor	0.08	0.9059
AKR1B10	-2.57	0.0002	aldo-keto reductase family 1, member B10	7q33	Metabolic enzyme	0.22	0.8146
PAIP2B	-2.50	0.0002	poly(A) binding protein interacting protein 2B	2p13.3	Transcription factor	0.70	0.2801
PIM2	-2.43	<0.0001	Pim-2 proto-oncogene, serine/threonine kinase	Xp11.23	Protooncogene	-0.26	0.5942
GPAT3	-2.26	0.0002	glycerol-3-phosphate acyltransferase 3	4q21.23	Metabolic enzyme	1.13	0.0679
XYLT2	-2.09	<0.0001	xylosyltransferase II	17q21.33	Metabolic enzyme	-0.15	0.7747
METTL7A	-2.09	0.0002	methyltransferase like 7A	12q13.12	Methyltransferase	-0.28	0.6188
FAM46C	-2.03	<0.0001	family with sequence similarity 46, member C	1p12	Translational factor	-0.28	0.5389
IL7R	-2.02	0.0002	interleukin 7 receptor	5p13	Cytokine receptor	-0.34	0.5839
BTG2	-1.83	0.0001	BTG family, member 2	1q32	Transcription factor	0.78	0.1001

GC tissue: primary gastric cancer tissue, Normal: corresponding adjacent normal gastric tissue, H-meta: hepatic metastasis tissue.

Supplementary Table 2. Primers and annealing temperatures

Abbreviations: MFSD4 major facilitator superfamily domain containing 4, *GAPDH* glyceraldehyde-3-phosphate dehydrogenase, *qRT-PCR* quantitative real-time reverse-transcription polymerase chain reaction.

Gene	Experiment	Type	Sequence (5´ - 3´)	Amplicon size	Annealing temperature
MFSD4	qRT-PCR	forward	CAACATGCAGCTGGTAAGGA	192 bp	60 °C
		reverse	ACCCTGGAGACATGGAACAG		
	Bisulfite sequencing	forward	AGTAGTTTTGTTTTTTGTTGGGTAG	299 bp	58 °C
		reverse	CCAAAAATCACCTTCAATCTCTAAA		
GAPDH	qRT-PCR	forward	GAAGGTGAAGGTCGGAGTC	226 bp	60 °C
		probe	CAAGCTTCCCGTTCTCAGCC		
		reverse	GAAGATGGTGATGGGATTTC		
